# Cerebral Oxygenation and Activity During Surgical Repair of Neonates With Congenital Diaphragmatic Hernia: A Center Comparison Analysis

**DOI:** 10.3389/fped.2021.798952

**Published:** 2021-12-17

**Authors:** Sophie A. Costerus, Dries Hendrikx, Joen IJsselmuiden, Katrin Zahn, Alba Perez-Ortiz, Sabine Van Huffel, Robert B. Flint, Alexander Caicedo, René Wijnen, Lucas Wessel, Jurgen C. de Graaff, Dick Tibboel, Gunnar Naulaers

**Affiliations:** ^1^Department of Paediatric Surgery, Erasmus Medical Center - Sophia Children's Hospital, Rotterdam, Netherlands; ^2^ESAT-STADIUS Division, Department of Electrical Engineering, KU Leuven, Leuven, Belgium; ^3^Department of Paediatric Surgery, University Hospital Mannheim, Mannheim, Germany; ^4^Neonatal Intensive Care Unit, University Hospital Mannheim, Mannheim, Germany; ^5^Division of Neonatology, Department of Paediatrics, Erasmus Medical Center - Sophia Children's Hospital, Rotterdam, Netherlands; ^6^Department of Pharmacy, Erasmus Medical Center, Rotterdam, Netherlands; ^7^Department of Anaesthesiology, Erasmus Medical Center, Rotterdam, Netherlands; ^8^Department of Development and Regeneration, KU Leuven, Leuven, Belgium; ^9^Neonatal Intensive Care Unit, University Hospitals Leuven, Leuven, Belgium

**Keywords:** neonates, surgery, midazolam, sevoflurane, cerebral activity, cerebral oxygenation

## Abstract

**Background and aim:** Neonatal brain monitoring is increasingly used due to reports of brain injury perioperatively. Little is known about the effect of sedatives (midazolam) and anesthetics (sevoflurane) on cerebral oxygenation (rScO_2_) and cerebral activity. This study aims to determine these effects in the perioperative period.

**Methods:** This is an observational, prospective study in two tertiary pediatric surgical centers. All neonates with a congenital diaphragmatic hernia received perioperative cerebral oxygenation and activity measurements. Patients were stratified based on intraoperatively administrated medication: the sevoflurane group (continuous sevoflurane, bolus fentanyl, bolus rocuronium) and the midazolam group (continuous midazolam, continuous fentanyl, and continuous vecuronium).

**Results:** Intraoperatively, rScO_2_ was higher in the sevoflurane compared to the midazolam group (84%, IQR 77–95 vs. 65%, IQR 59–76, *p* = < 0.001), fractional tissue oxygen extraction was lower (14%, IQR 5–21 vs. 31%, IQR 29–40, *p* = < 0.001), the duration of hypoxia was shorter (2%, IQR 0.4–9.6 vs. 38.6%, IQR 4.9–70, *p* = 0.023), and cerebral activity decreased more: slow delta: 2.16 vs. 4.35 μ*V*^2^ (*p* = 0.0049), fast delta: 0.73 vs. 1.37 μ*V*^2^ (*p* = < 0.001). In the first 30 min of the surgical procedure, a 3-fold increase in fast delta (10.48–31.22 μ*V*^2^) and a 5-fold increase in gamma (1.42–7.58 μ*V*^2^) were observed in the midazolam group.

**Conclusion:** Sevoflurane-based anesthesia resulted in increased cerebral oxygenation and decreased cerebral activity, suggesting adequate anesthesia. Midazolam-based anesthesia in neonates with a more severe CDH led to alarmingly low rScO_2_ values, below hypoxia threshold, and increased values of EEG power during the first 30 min of surgery. This might indicate conscious experience of pain. Integrating population-pharmacokinetic models and multimodal neuromonitoring are needed for personalized pharmacotherapy in these vulnerable patients.

**Trial Registration:** https://www.trialregister.nl/trial/6972, identifier: NL6972.

## Introduction

Clinicians are increasingly monitoring the neonatal brain during high-risk neonatal surgery, due to the increased number of neonates who develop brain injury perioperatively, and subsequently impaired neurodevelopmental outcome (1–4).

Multimodal brain monitoring is needed to monitor the interplay between cerebral oxygenation and activity (5). Near-infrared spectroscopy (NIRS) presents a continuous, non-invasive measurement of regional cerebral oxygen saturation (rScO_2_), with reference values between 55 and 85% for awake neonates (6). Baseline rScO_2_ can vary as a result of sensor placement (7, 8), sensor type (9, 10), and the measurement device (11, 12). Furthermore, it is possible to analyse cerebral oxygen consumption by using the fractional tissue oxygen extraction (FTOE) (13). Intraoperative use of NIRS has been explored, but no coherent results have been reported in non-cardiac neonatal surgery (5) and NIRS-guided treatment guidelines are only available in pediatric cardiac surgery (14).

The activity of the neonatal brain is quantified using electroencephalography (EEG), which measures the overall electrical activity of the cortical pyramidal neurons (15). Consequently, EEG has a great sensitivity of showing changes in neural functioning (16). The power of the EEG is computed in different frequency bands. Delta oscillations (δ: 0.5–4 Hz) dominate the neonatal EEG and regulate basic homeostatic need (17), which can be divided into slow delta (δ_1_: 0.5–2 Hz) or fast delta (δ_2_: 2–4 Hz). Noxious-evoked EEG activity can be studied by analyzing gamma oscillations (γ: 32–100 Hz) over the contralateral somatosensory cortex and by analyzing the fast delta band (δ_2_) (18). Strong increased gamma γ oscillations and increased energy in the fast delta δ_2_ band was shown to reflect nociceptive pain in neonates following heel lance (18).

To date, little is known about the effect of anesthetic approaches on cerebral oxygenation (5, 19). In this prospective center comparison study, neonates with a congenital diaphragmatic hernia (CDH), treated according to a standardized international guideline (20) received two different anesthetic approaches intraoperatively: midazolam with analgesia and muscle relaxation vs. an sevoflurane with analgesia and muscle relaxation. We hypothesize that cerebral oxygenation is affected to a lesser extent by the administration a sedative-agent (midazolam), compared to the administration of anesthetic-agent (sevoflurane), assuming that the cerebral activity is less affected by a sedative-agent compared to an anesthetic-agent. The aim of this study was to determine the effects of midazolam vs. sevoflurane on cerebral oxygenation and cerebral activity in the perioperative period.

## Materials and Methods

This is an observational, prospective center comparison study on perioperative neuromonitoring in neonates with a CDH. All patients underwent surgery in a tertiary pediatric referral center: the Sophia Children's Hospital (Rotterdam, the Netherlands), or the Mannheim University Hospital (Mannheim, Germany), which treat ~20 and 60 neonates a year, respectively. Local institutional review board approval (Erasmus MC, Rotterdam, The Netherlands, MEC-2017–145, and Universitätsmedizin Mannheim, Mannheim, Germany 2018-578N-MA) and written informed consent of parents or legal guardians were obtained. The study is registered in the Netherlands trial register (www.trialregister.nl), clinical trial number NL6972.

### Patients

This study focused on neonates with a CDH, a major non-cardiac anomaly, that requires surgical repair within the 1st days of life. CDH neonates were eligible for inclusion if surgical repair was scheduled between July 2018 and April 2020 within the first 28 days of life, regardless of the type of surgery (open or thoracoscopic surgery), or the need for extracorporeal membrane oxygenation (ECMO) therapy until 24 h before surgery, for which both centers used the same entry criteria (20). Neonates were excluded if they had major cardiac or chromosomal anomalies, syndromes associated with altered cerebral perfusion, or syndromes associated with major neurodevelopmental impairment. In addition, neonates on ECMO during the start of the procedure were also excluded. Patients were treated according to the CDH-EURO consortium guidelines (20).

### Perioperative Management

The neonates enrolled in this study were stratified in two groups. In Rotterdam, surgery was performed in the operating room (OR) and anesthesia was sevoflurane based (end expired concentration between 1 and 2%), with bolus fentanyl (induction 1–5 ug kg^−1^) and rocuronium (0.5–1.0 mg kg^−1^) performed by a pediatric anaesthesiologist. Patients operated at the OR underwent thoracoscopic surgery if they were hemodynamically stable and if the liver was not herniated. In Mannheim, surgery was performed at the neonatal intensive care unit (NICU), and anesthesia was based on continuous midazolam (70–100 ug kg^−1^ h^−1^), fentanyl (2–5 ug kg^−1^ h^−1^), vecuronium (0.10–0.30 mg kg^−1^), and bolus fentanyl (2–10 ug kg^−1^), performed by a neonatologist/ECMO specialist according to center specific routine for many years. Performing surgery in the NICU was partly based on the cardiorespiratory instability of the patient. Repeated administration of analgesia was based on clinical evaluation.

### Data Collection

Patient demographics were collected according to the international consensus about standardized reporting for CDH (21). Measurements were performed in a continuous fashion, started at least 6 h before surgery and continued up to 24 h after surgery. Perioperative management, such as the administration of medication and the analysis of arterial blood gasses, was recorded in the patient data management system (HiX, Chipsoft BV, Amsterdam, the Netherlands).

Heart rate (HR), intra-arterial mean arterial blood pressure (MABP) and peripheral arterial oxygen saturation (SpO_2_) were measured at 1 Hz (Primus, Draeger, Luebeck, Germany). rScO_2_ was measured at a sampling rate of 1 Hz using NIRS (neonatal sensor, INVOS 5100C, Covidien, Boulder, Colorado, United States) with a hypoxic threshold of 63% for this device-sensor combination (10, 11). EEG was recorded using a 4-channel EEG at 256 Hz (Rotterdam: BrainRT, OSG, Rumst, Belgium, Mannheim: Braintrend, Fritz Stephan GMBH, Gackenbach, Germany). The power of the EEG was computed in the slow delta (δ_1_: 0.5–2 Hz), fast delta (δ_2_: 2–4 Hz), and gamma (γ: 32–100 Hz) frequency bands. Clinicians were blinded for EEG, but not for rScO_2_.

In Rotterdam, the Shell+ RT software Suite of the BrainRT was reprogrammed for real-time data extraction. In Mannheim, AnStat software (Carepoint, Ede, the Netherlands) was used for real-time data extraction. Both software extracted data in the same manner. The vasoactive-inotropic score (VIS) reflects the need and grade of vasoactive/inotropic pharmaceutical intervention and was calculated to quantify necessity of cardiovascular support (22).

### Data Processing and Statistical Analysis

The signal processing pipeline started with three pre-processing steps. First, artifacts were detected and removed. For EEG, segments were excluded from the analysis if the absolute amplitude exceeded 500 μ*V*, which is the maximum voltage that could be detected by the monitor. For the other signals, segments outside of the physiological range, segments containing motion artifacts were removed. Second, the spectral power in the EEG was computed using the continuous wavelet transform and ridge extraction (23). Three frequency bands of interest were defined: slow delta δ_1_ (0.5–2 Hz), fast delta δ_2_ (2–4 Hz), and gamma γ (30–100 Hz) frequency bands. Third, FTOE was computed from rScO_2_ and SpO_2_ values as FTOE = (SpO_2_ – rScO_2_)/SpO_2_. Since rScO_2_ mainly reflects the oxygenation of the venous return from the brain, FTOE defines the amount of oxygen extracted in the brain.

After these processing steps, signal parameters were extracted in four data windows: the *preoperative* window (Pr: 6–3 h before the start of surgery), the *intraoperative* window (In: entire surgical procedure), and two *postoperative* windows (Po3: 3–6 h and Po15: 15–18 h after the end of surgery). These data windows were used to balance the data and to remove transitional effects, such as artifacts of transport, artifacts of care, the effect of intraoperative administered medication.

*T*-test was used for the comparison of the patient demographics. Generalized least-squares models were used for the statistical analysis of the primary endpoint, since they showed to be the best fit for the data as indicated by the Akaike information criterion (24). *Post-hoc* comparisons were based on analysis of marginal means, implemented with Tukey's correction for multiple comparisons. All statistical computations were carried out in R, with significance defined as α < 0.05 (25).

### Correlations

Three correlations were studied to determine their effect on our results. Firstly, the correlation between vasoactive and inotropic medication on rScO_2_ and FTOE was studied to clarify possible effects of cerebral vasoconstriction. Secondly, the correlation between the dosage of anesthesia and cerebral oxygenation and activity was studied to clarify dosage related changes in rScO_2_ and EEG-power values. Thirdly, the correlation between cerebral oxygenation and activity was investigated to study whether the assumption of reduced cerebral activity resulting in increased rScO_2_ holds true in this study.

### Endpoints

The primary endpoint of this study was measurement of the differential effect of sevoflurane vs. midazolam on cerebral oxygenation perioperatively. The secondary endpoint of this study was measurement of the differential effect of sevoflurane vs. midazolam on cerebral activity and the vital parameters perioperatively.

The primary endpoint of this study was measurement of the differential effect of sevoflurane vs. midazolam on cerebral oxygenation perioperatively. The secondary endpoint of this study was measurement of the differential effect of sevoflurane vs. midazolam on cerebral activity and the vital parameters perioperatively.

## Results

### Demographics

Informed consent was obtained in 49 CDH neonates. Five patients were excluded following the absence of multiple signals due to data transfer and/or storage problems, six patients were excluded due to the need of extracorporeal membrane oxygenation (ECMO) treatment and one patient was excluded due to cardiopulmonary resuscitation intra-operatively. Therefore, 37 neonates could be analyzed: 20 neonates in the sevoflurane group (all patients from Rotterdam and received surgery in the OR), 17 neonates in the midazolam group (all patients from Mannheim and received surgery in the NICU) (Appendix 1). In the midazolam group, five neonates received VA-ECMO treatment until 1 day before surgery. Both groups were comparable (Table 1), except for a lower amount of herniated livers (25 vs. 71%, *p* = 0.005), a lower VIS preoperatively (0, IQR 0–5 vs. 17, IQR 10–25, *p* = < 0.001), a lower postnatal age on the day of surgery (3 days, IQR 2–4 vs. 6 days, IQR 3–12, *p* = 0.008), more thoracoscopic surgery (40 vs. 23%, *p* = 0.026), and shorter surgery (95 min, IQR 70–125 vs. 182 min, IQR 114–203, *p* = < 0.001) in the sevoflurane group compared to the midazolam group. Eight patients who underwent thoracoscopic surgery were converted to an open approach because of the need for a patch.

**Table 1:** Patient demographics of the sevoflurane and the midazolam group.

	**Sevoflurane group**	**Midazolam group**	***p*-value**
*N*	20	17	
Male	55% (11)	59% (10)	0.821
Gestational age (wk)	38+1 (36+5 – 38+5)	37+6 (34+5 – 38+1)	0.111
Birth weight (kg)	3.0 (2.7–3.3)	2.8 (1.9–3.1)	0.070
Apgar 5 min	8 (8,9)	8 (7-8)	0.478
o/e LHR	50 (41–58)	40 (33-54)	0.337
Preoperative mechanical ventilation (%)	85% (17)	100%	0.101
Preoperative VA-ECMO	0%	29% (5)	0.022
Intraoperative VA-ECMO	0%	0%	1.00
Left sided defect (%)	85% (17)	65% (11)	0.977
Liver-up	25% (5)	71% (12)	0.005
Age at surgery (d)	3 (2–4)	6 (3-12)	0.008
Thoracoscopy/laparotomy	40/60% (8/12)	23/77% (4/13)	0.026
Conversion	100% (8)	0%	0.002
Duration of surgery (min)	95 (70–125)	182 (114–203)	0.000
Defect size (*n*)	6A, 8B, 5C, 1D	1A, 9B, 6C,1D	0.185
Patch (%)	60% (12)	88% (15)	0.056
VIS-score preoperative	0 (0–5)^$^	17 (10–25)^$^	0.000
VIS-score intraoperative	9 (5-25)^$^	17 (12–35)^$^	0.010
VIS-score postoperative (ug/kg)	2 (0–11)^$^	17 (10–28)^$^	0.001
Rocuronium bolus dosage intraoperative (mg/kg)	0.8 (0.6–1.0)	-	
Vecoronium bolus dosage during induction (mg/kg)	-	0.2 (0.15–0.21)	
Vecoronium perfusor dosage intraoperative (mg/kg/h)	-	0.09 (0.05–0.10)	
Fentanyl bolus dosages during induction (ug/kg)	2.3 (1.7–2.9)	5 (4–7)	0.000
Cumulative fentanyl bolus dosages intraoperative (ug/kg/h)	6.2 (4.1–11.5)	10 (7–28)	0.119
Fentanyl perfusor dosages intraoperative (ug/kg/h)	-	4 (3–5)	
Sevoflurane concentration [end expired concentration (%)]	1.5 (1.1–1.9)^*^	-	-
Midazolam perfusor dosage preoperative (ug/kg/h)	47 (0–92)	40 (30–50)	0.735
Midazolam perfusor dosage intraoperative (ug/kg/h)	47 (0–67)	100 (68–100)	0.003
Midazolam perfusor dosage postoperative (ug/kg/h)	39 (26–99)	50 (20–50)	0.647
Midazolam bolus intraoperative (%)	5% (1)	59% (10)	0.000

$ *Data presented as median (IQR) or (range)*.

* *Range of the mean values*.

### Anesthesia

Preoperatively, in the sevoflurane group, five neonates were not sedated while on mechanical ventilation, two neonates received continuous administration of morphine, seven neonates received continuous administration of midazolam, and six neonates received continuous administration of both midazolam and morphine (Appendix 2). In the midazolam group, all neonates received continuous midazolam, supplemented with continuous administration of fentanyl (Appendix 2) and were intubated multiple days before surgery. The patients in the sevoflurane group that were intubated and sedated before start of surgery received comparable dosages of midazolam (47 ug kg^−1^ h^−1^, IQR 0–92 and 40 ug kg^−1^ h^−1^, IQR 30–50, *p* = 0.735, respectively, Table 1) compared to the midazolam group.

Intraoperatively, the end expired sevoflurane concentration was 1.5% (IQR 1.1–1.9). Time between the start of administration of sevoflurane and start of the surgical procedure was 66 min (IQR 45–76.6). In six neonates, the preoperative continuous midazolam administration was continued intraoperatively and in two neonates the preoperative continuous morphine administration was continued intraoperatively during sevoflurane anesthesia (Appendix 2). Three neonates received a bolus of propofol (2.9, 3.7, and 7.3 mg kg^−1^ for endotracheal intubation). In the midazolam group, the midazolam dosage was 100 ug kg^−1^ h^−1^ (IQR 68–100). The time between the administration of midazolam and the start of the surgical procedure was 21 min (IQR 1–30). The midazolam dosage in the sevoflurane group of those in whom the midazolam was continued from PICU (*n* = 6) was significantly lower (47 ug kg^−1^ h^−1^, IQR 0–67, *p* = 0.003) compared to the midazolam group (100 ug kg^−1^ h^−1^, IQR 68–100) (Table 1). The fentanyl bolus dosage during the induction of anesthesia was lower in the sevoflurane group compared to the midazolam group (2 ug kg^−1^, IQR 2–3 vs. 5 ug kg^−1^, IQR 4–7 vs. *p* = < 0.001), although the cumulative fentanyl bolus dosages that were administrated intraoperatively did not differ (10 ug kg^−1^, IQR 7–17 vs. 6 ug kg^−1^, IQR 4–12, *p* = 0.119). Yet, the midazolam group received additional continuous administration of fentanyl (4 ug kg^−1^ h^−1^, IQR 3–5), whereas the sevoflurane group did not.

Postoperatively, the midazolam dosages did not differ significantly between the sevoflurane (39 ug kg^−1^ h^−1^, IQR 26–99) and the midazolam group (48 ug kg^−1^ h^−1^, IQR 20–50) (Table 1 and Appendix 2).

### Vital Parameters

**Figure 1:**
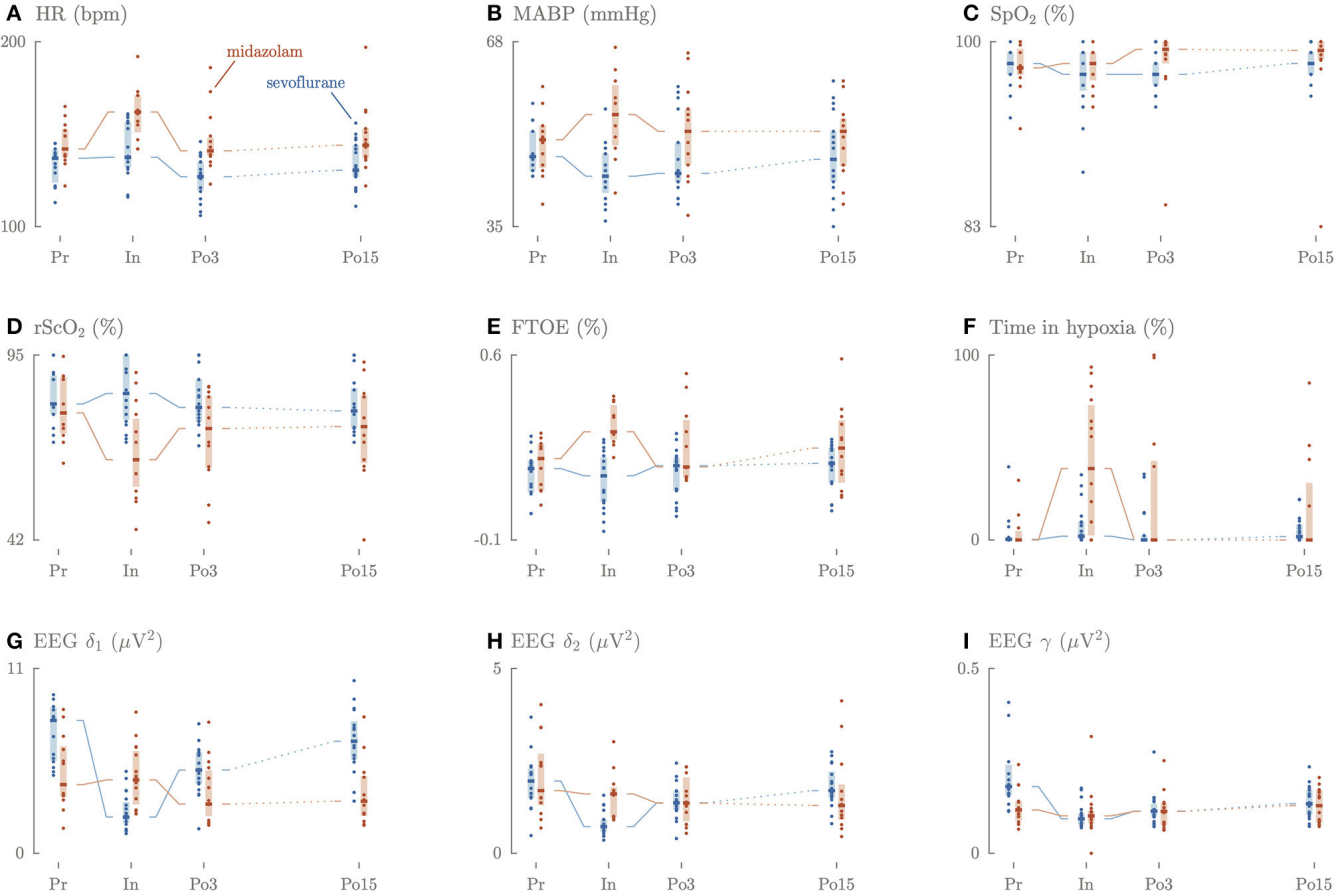
Longitudinal overview of the perioperative changes in HR **(A)**, MABP **(B)**, SpO_2_ **(C)**, rSO_2_ **(D)**, FTOE **(E)**, time in hypoxia **(F)**, slow delta **(G)**, fast delta **(H)**, gamma **(I)** power.

Preoperatively, HR was significantly lower in the sevoflurane group (137 bpm, IQR 126–141) compared to the midazolam group (142 bpm, IQR 138–152) (Figure 1A, Appendix 3). MABP did not differ, although the VIS was significantly lower in the sevoflurane group (0, IQR 0–5) than in the midazolam group (17, IQR 10–25). Intraoperatively, HR and MABP significantly dropped in sevoflurane group (HR: 138 bpm, IQR 132–156 and MABP: 44 mmHg, IQR42–48) compared to the midazolam group (HR: 162 bpm, IQR 153–171 and 55 mmHg, IQR 50–60). Intraoperatively, the VIS score was again significantly lower in the sevoflurane group (9, IQR 5–17) than in the midazolam group (17, IQR 12–35).

Three until 6 h postoperatively, HR remained significantly lower in the sevoflurane group (127 bpm, IQR 120–135) compared to the midazolam group (141 bpm, IQR 139–148). VIS was still significantly lower in the sevoflurane group (2, IQR 0–11) compared to the midazolam group (17, IQR 10–28).

Fifteen until 18 h postoperatively, HR remained significantly lower in the sevoflurane group (131 bpm, IQR 127–135) compared to the midazolam group (144 bpm, IQR 137–153). SpO_2_ and PaCO_2_ did not differ between the group perioperatively.

### Cerebral Oxygenation

Preoperatively, rScO_2_, FTOE and duration of cerebral hypoxia did not differ significantly between the groups (Figure 1).

Intraoperatively, the rScO_2_ values were significantly higher in the sevoflurane group (84%, IQR 77–95) compared to the midazolam group (65%, IQR 59–76, *p* < 0.001). The opposite was true for FTOE, which was lower in the sevoflurane group (14%, IQR 5–21) compared to the midazolam group (31%, IQR 29–40, *p* < 0.001). The duration of hypoxia was significantly shorter in the sevoflurane group (2%, IQR 0.4–9.6) compared to the midazolam group (38.6%, IQR 4.9–70, *p* = 0.023).

Three until 6 h and 15 until 18 h postoperatively, rScO_2_, FTOE, and the duration of cerebral hypoxia did not differ between the groups.

### Cerebral Activity

Preoperatively, power in the EEG slow delta δ_1_ and gamma γ frequency bands was significantly higher in the sevoflurane group (7.9 μ*V*^2^, IQR 5.5–8.6; 0.19 μ*V*^2^, IQR 0.16–0.28) compared to the midazolam group (4.1 μ*V*^2^, IQR 3.4–6.2, *p* = 0.009 vs. 0.12 μ*V*^2^, IQR 0.09–0.14, *p* = 0.0017), but not for the fast delta δ_2_ frequency (2.0 μ*V*^2^, IQR 1.5–2.3 vs. 1.7 μ*V*^2^, IQR 1.4–2.5) (Figure 1).

**Figure 2:**
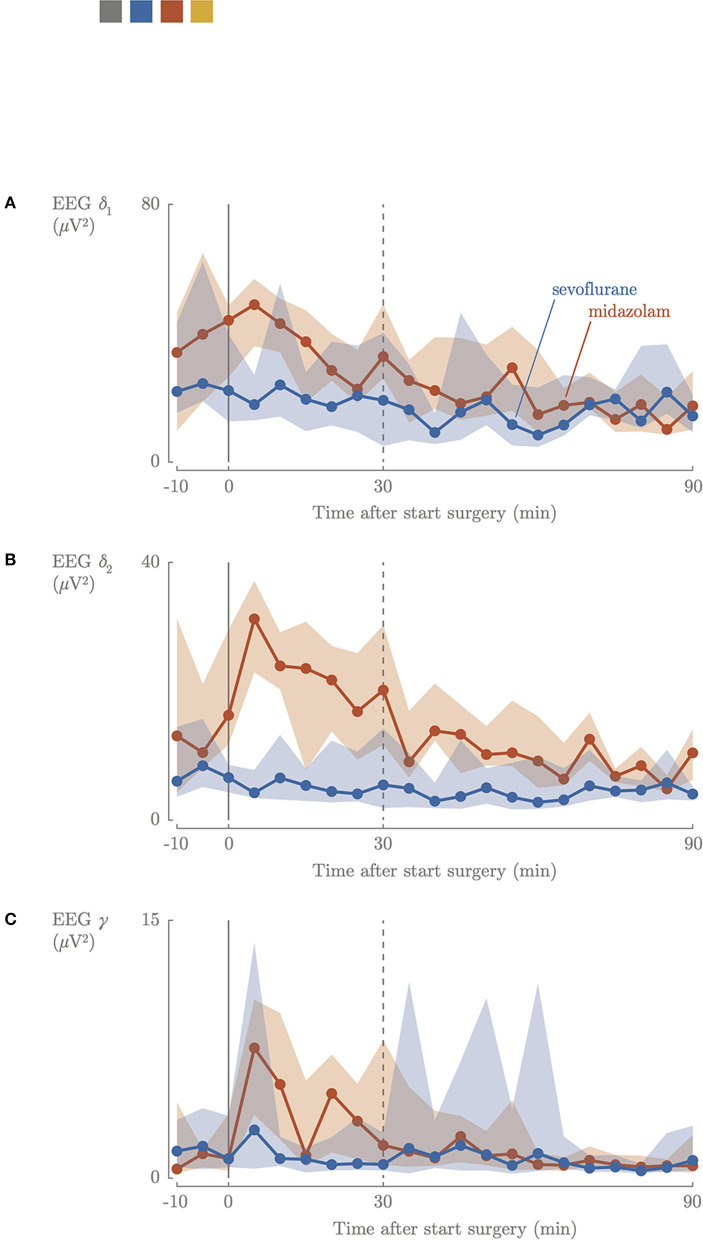
Intraoperative cerebral activity; slow delta **(A)**, fast delta **(B)**, gamma **(C)** power. Blue, sevoflurane group; red, midazolam group; 0, start surgical procedure.

Intraoperatively, slow delta δ_1_, fast delta δ_2_ and gamma γ power decreased in the sevoflurane group and remained decreased during the entire intraoperative period. In the midazolam group a 3-fold increase of fast delta δ_2_ power was observed in the first 30 min of the surgical procedure, which decreased later on. A comparable increase was observed for the gamma γ frequency, which was characterized by a 5-fold increase (Figure 2). The intraoperative median values of slow delta δ_1_*and* fast delta δ_2_ power decreased significantly in the sevoflurane group (2.2 μ*V*^2^, IQR 1.9–3.0, 0.73 μ*V*^2^, IQR 0.59–0.91) compared to the midazolam group (4.4 μ*V*^2^, IQR 3.1–6.0, *p* = 0.0002, 1.6 μ*V*^2^, IQR 1.0–1.7, *p* = < 0.001), but not for gamma γ power (0.09 μ*V*^2^, IQR 0.08–0.10, 0.10 μ*V*^2^, IQR 0.08–0.11).

**Figure 3:**
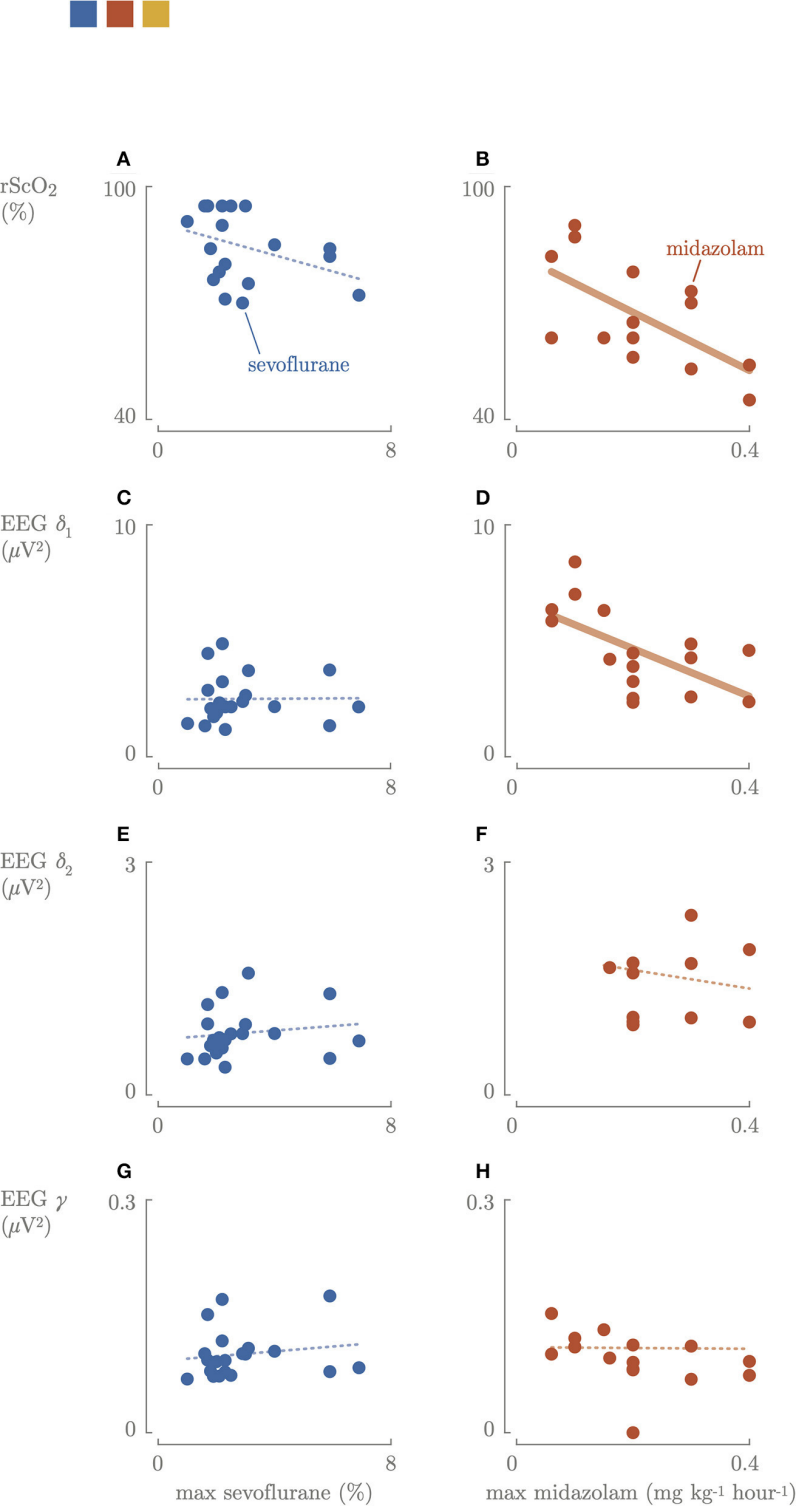
Correlation between the maximum sevoflurane concentration or maximum dosages of midazolam cerebral oxygenation **(A,B)** and cerebral activity: slow delta **(C,D)**, fast delta **(E,F)**, gamma **(G,H)** power. Blue, sevoflurane group; red, midazolam group; fat line, significant correlation.

Higher maximal sevoflurane concentration (end expired concentration) was not associated with lower cerebral activity (Figure 3). Higher maximum dosages of midazolam were associated with lower cerebral activity (*p* = 0.023) (Figure 3). The neonates who received a bolus of propofol had EEG delta frequency power (2.15–2.16 μ*V*^2^) comparable to the other neonates in the sevoflurane group. Three until 6 h postoperatively, the EEG slow delta δ_1_, fast delta δ_2_, and gamma γ frequencies increased again in the sevoflurane group (5.0 μ*V*^2^, IQR 4.2–6.0, 1.4 μ*V*^2^, IQR 1.2–1.7, 0.11 μ*V*^2^, IQR 0.10–0.13), decreased in the midazolam group (2.9 μ*V*^2^, IQR 2.4–4.8, 1.36 μ*V*^2^, IQR 0.95–1.93, 0.11 μ*V*^2^, IQR 0.08–0.13) compared to the intraoperative period.

Fifteen until 18 h postoperatively, the EEG slow delta δ_1_, fast delta δ_2_, and gamma γ frequencies increased further in the sevoflurane group (6.7 μ*V*^2^, IQR 5.6–7.6, 1.7 μ*V*^2^, IQR 1.5–2.4, 0.14 μ*V*^2^, IQR 0.10–0.17), increased in the midazolam group (3.1 μ*V*^2^, IQR 2.4–4.8, 1.31 μ*V*^2^, IQR 0.98–2.23, 0.13 μ*V*^2^, IQR 0.08–0.17) compared to the intraoperative period.

### Correlations

**Figure 4:**
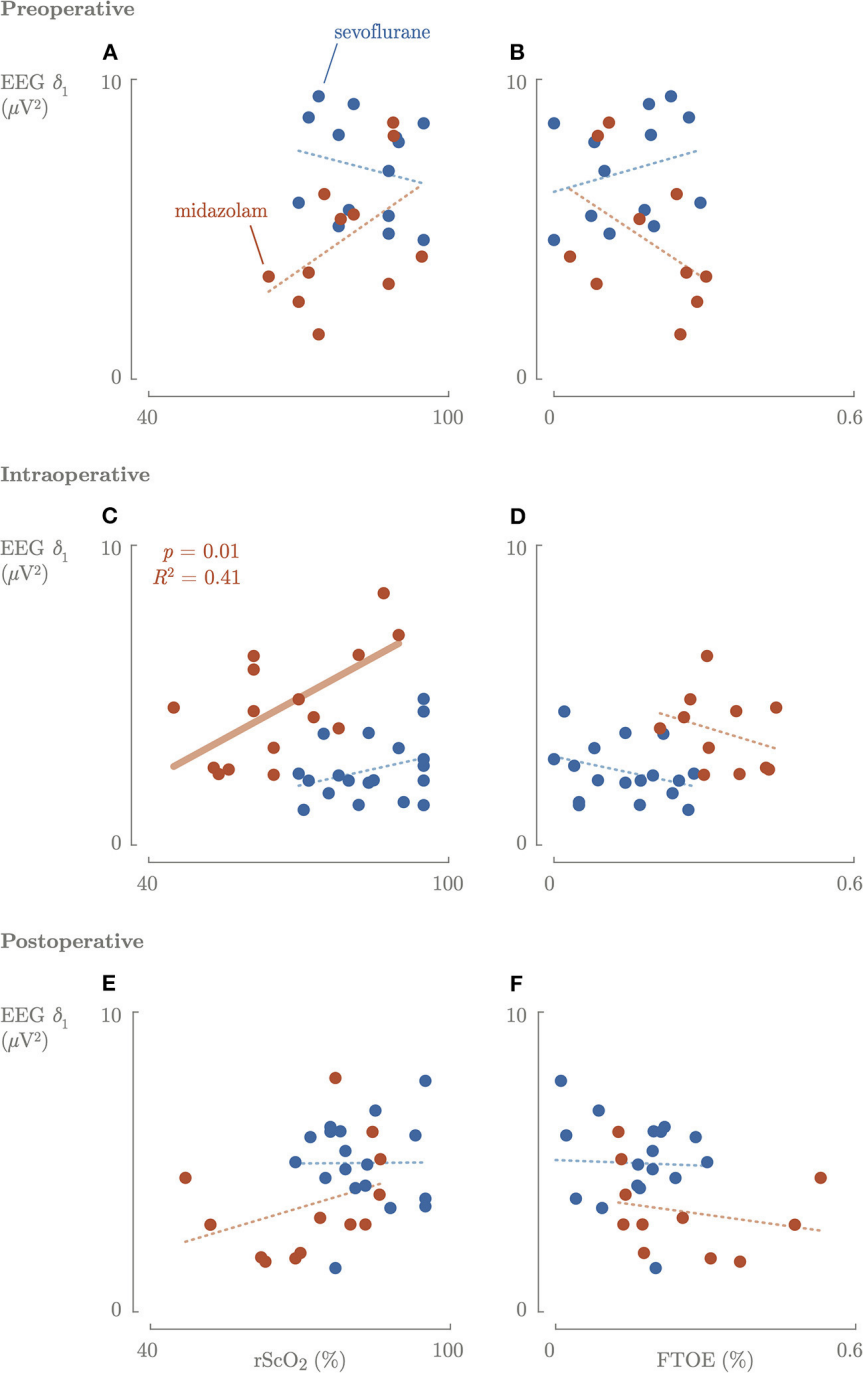
Correlation between the cerebral activity and cerebral oxygenation and oxygen consumption preoperative **(A,B)**, intraoperative **(C,D)**, and postoperative **(E,F)**. Blue, sevoflurane group; red, midazolam group; fat line, significant correlation.

Preoperative, no correlation between rScO_2_ or FTOE and cerebral activity was observed (Figure 4).

Intraoperatively, a significantly (*p* = 0.01) positive correlation between rScO_2_ and cerebral activity in the midazolam group was found (Figure 4). In contrast, there was no correlation between FTOE and cerebral activity. Higher maximum dosages of midazolam resulted in a significant decrease in rScO_2_ and slow delta EEG power (Figure 3). The same trend was observed for maximum sevoflurane concentration, although not significant. In the sevoflurane group, rScO_2_ was negatively correlated with VIS (*R*^2^ = 0.23, *p* = 0.04) and positively correlated with FTOE (*R*^2^ = 0.21, *p* = 0.04). These correlations were not found in the midazolam group.

Postoperatively, rScO_2_ was negatively correlated with VIS (*R*^2^ = 0.35, *p* = 0.01) and FTOE positively correlated with VIS (*R*^2^ = 0.32, *p* = 0.001) in the sevoflurane group, and not in the midazolam group.

## Discussion

In our study surgery during sevoflurane-based anesthesia resulted in stable cerebral oxygenation, decreased oxygen consumption and decreased cerebral activity. The EEG power did not indicate pain stimuli awareness. Surgery during midazolam-based anesthesia resulted in low rScO_2_, increased cerebral oxygen consumption and increased cerebral activity throughout the first 30 min of the surgical procedure. The increased EEG power of the fast delta and the gamma band indicated conscious pain perception.

Intraoperatively, rScO_2_ values were observed to be significantly higher in the sevoflurane group compared to the midazolam group, which reached alarmingly low rScO_2_ values (Figure 1). In the sevoflurane group, we observed a stable rScO_2_ and a decreased FTOE, despite a reduction in MABP (Figure 1), which is in line with a study in children between 0 and 2 years (26). In the midazolam group, a decrease in rScO_2_ and an increase in FTOE were observed, despite an increase in MABP and HR (Figure 1). Vascular resistance and subsequently cerebral perfusion are affected by the partial pressure of carbon dioxide (PaCO_2_) and by vasoactive and inotropic medication (27, 28). In this study, both groups had comparable PaCO_2_ levels, which were stable and within clinical range. In the sevoflurane group, increased VIS was associated with lower rScO_2_ values and higher FTOE intra- and postoperatively, which might reflect increased vascular resistance. Yet, the hemodynamic support, mostly with norepinephrine, was the highest in the midazolam group (Table 1), without affecting rScO_2_ or FTOE. A recent study signals that norepinephrine elevates MABP, while reducing cerebral perfusion in adults (29). This is in line with our result in the sevoflurane group.

A recent study showed that higher sevoflurane doses significantly correlated with more suppressed background patterns (30). This correlation was not observed in our study, but this could be due to the relatively small range in sevoflurane concentrations that were administered (Figure 3). Overall, EEG power didn't decrease during midazolam-based anesthesia, but increased midazolam dose was found to be associated with a decrease in brain activity. The median time between bolus administration or increased perfusor dosages of midazolam and the start of surgical procedure was 21 min. During the first 30 min of surgery, a 3-fold increase in δ_2_ power and a 6-fold increase in gamma γ power were observed, suggesting that nociceptive stimuli were registered by the brain (Figure 2) (18). After 30 min, the power in the different EEG bands stabilized to lower values. This might indicate that the increase in midazolam dose requires (at least) 21 plus 30 min to reach a new steady state concentration, and/or that surgery started too soon after administering midazolam and analgesia, since cerebral activity decreased after 30 min. A loading dose helps to induce the effect quicker, although this was given in 59% of the neonates in the midazolam group.

Patients in the midazolam group received midazolam preoperatively for multiple days and had a median midazolam dosage of 0.1 mg kg^−1^h^−1^ intraoperatively. This is substantially higher than the dosing advice of 0.06 mg kg^−1^h^−1^ for sedation with midazolam in neonates with a gestational age above 32 weeks (31), although this dosing advice was already questioned by our research group (32). The elimination half-life of midazolam is ~6 h in the 1st week of life in full-term neonates (33), although severity of disease and inflammation may also affect the elimination of midazolam in critically ill neonates (34). A recent Cochrane review concluded that midazolam was an effective sedative in neonates (35), although transient cerebral hypoperfusion was observed after a bolus of midazolam, as well as significant higher rates of adverse neurological events in neonates treated with midazolam compared to morphine (36, 37). In this study, neonates in the midazolam group all received continuous administration of fentanyl. Single use of fentanyl dosages of 50–100 ug kg^−1^ is a commonly used anesthetic approach during congenital cardiac surgery or if the neonate has limited hemodynamic reserve (38). A randomized trial in 1987 already proved the additional value of fentanyl in the stress response of neonates intraoperatively (39). Another study compared the effect of fentanyl with fentanyl plus midazolam on stress response during neonatal cardiac surgery, and concluded that intraoperative administration of fentanyl plus midazolam did not reduce stress response compared fentanyl (38). This suggests that high doses of fentanyl might be a better anesthetic approach than midazolam with lower dosages of fentanyl.

The strengths of this study are that both centers have long-lasting experience treating high-risk CDH patients and have acted as “founding fathers” for well-established international guidelines. In addition, neither the treatment modalities, nor the composition of the treatment teams changed during the study period. Both centers used validated pain assessment instruments (Comfort-B) as pharmacodynamic endpoint to evaluate and treat pain.

This study has two main limitations. First, exposure to medication was compared based on administered dosages instead of plasma concentrations, following the strict rules on blood sampling in neonates. Second, this is a center comparison study and not a randomized controlled trial. In this study, the neonates in the midazolam group were more critically ill. The neonates in the midazolam group were operated in the NICU, partly because of the preference of the clinicians and partly because of cardiorespiratory instability of the patient. In this group, the defect of the diaphragm was more severe (more patient with a herniated liver) and the neonates in this group needed more hemodynamic support preoperatively (higher VIS). Intraoperatively, however, the hemodynamic support did not differ between the midazolam and the sevoflurane group. In addition, we found a negative correlation between rScO_2_ and VIS and a positive correlation between rScO_2_ and FTOE in the sevoflurane group and not in the midazolam group. Furthermore, more critically ill patients may have a longer elimination time of midazolam.

## Conclusion

This comparison of two approaches for CDH surgery showed that sevoflurane-based anesthesia resulted in increased cerebral oxygenation and decreased cerebral activity, suggesting adequate anesthesia. Midazolam-based anesthesia in neonates with a more severe CDH (more liver-up and higher VIS) led to alarmingly low rScO_2_ values, below hypoxia threshold, and increased values of EEG power during the first 30 min of surgery. This might indicate conscious experience of pain.

These results stimulate the integration of population-pharmacokinetic models in combination with multimodal neuromonitoring to reach evidence-based perioperative pharmacotherapy in these vulnerable patients.

## Data Availability Statement

The raw data supporting the conclusions of this article will be made available by the authors, without undue reservation.

## Ethics Statement

The studies involving human participants were reviewed and approved by Erasmus MC, Rotterdam, The Netherlands, MEC-2017–145, and Universitätsmedizin Mannheim, Mannheim, Germany 2018-578N-MA. Written informed consent to participate in this study was provided by the participants' legal guardian/next of kin.

## Author Contributions

SC and DH: substantial contributions to the conception or design of the work, the acquisition, analysis, interpretation of data for the work, drafting the work, final approval of the version to be published, and agreed to be accountable for all aspects of the work in ensuring that questions related to the accuracy or integrity of any part of the work are appropriately investigated and resolved. JI, KZ, AP-O, SV, RF, AC, RW, LW, JG, DT, and GN: substantial contributions to the conception and design of the work, analysis, interpretation of data for the work, revising it critically for important intellectual content, final approval of the version to be published, and agreed to be accountable for all aspects of the work in ensuring that questions related to the accuracy or integrity of any part of the work are appropriately investigated and resolved. All authors contributed to the article and approved the submitted version.

## Funding

Research supported by Bijzonder Onderzoeksfonds KU Leuven (BOF): C24/15/036. The effect of perinatal stress on the later outcome in preterm babies, EU: H2020 MSCA-ITN-2018: Integrating Functional Assessment measures for Neonatal Safeguard (INFANS), funded by the European Commission under Grant Agreement #813483. This research received funding from the Flemish Government (AI Research Program). SV and DH are affiliated to Leuven. AI—KU Leuven Institute for AI, B-3000, Leuven, Belgium. DH was a SB Ph.D. fellow at Fonds voor Wetenschappelijk Onderzoek (FWO), Vlaanderen, supported by the Flemish government.

## Conflict of Interest

The authors declare that the research was conducted in the absence of any commercial or financial relationships that could be construed as a potential conflict of interest.

## Publisher's Note

All claims expressed in this article are solely those of the authors and do not necessarily represent those of their affiliated organizations, or those of the publisher, the editors and the reviewers. Any product that may be evaluated in this article, or claim that may be made by its manufacturer, is not guaranteed or endorsed by the publisher.

## Supplementary Material

Appendix 1 Flowchart of the included patients.

Appendix 2 Overview of administrated medication in the perioperative period.

Appendix 3 Overview of exact values Pr, In, Po3.
