# Supplementary figures and images for: Cerebral Oxygenation and Activity During Surgical Repair of Neonates With Congenital Diaphragmatic Hernia: A Center Comparison Analysis
Source: Front Pediatr. 2021 Dec 17;9:798952. doi: 10.3389/fped.2021.798952 (PMC8718750; doi:10.3389/fped.2021.798952)

Preoperative

Intraoperative

Postoperative

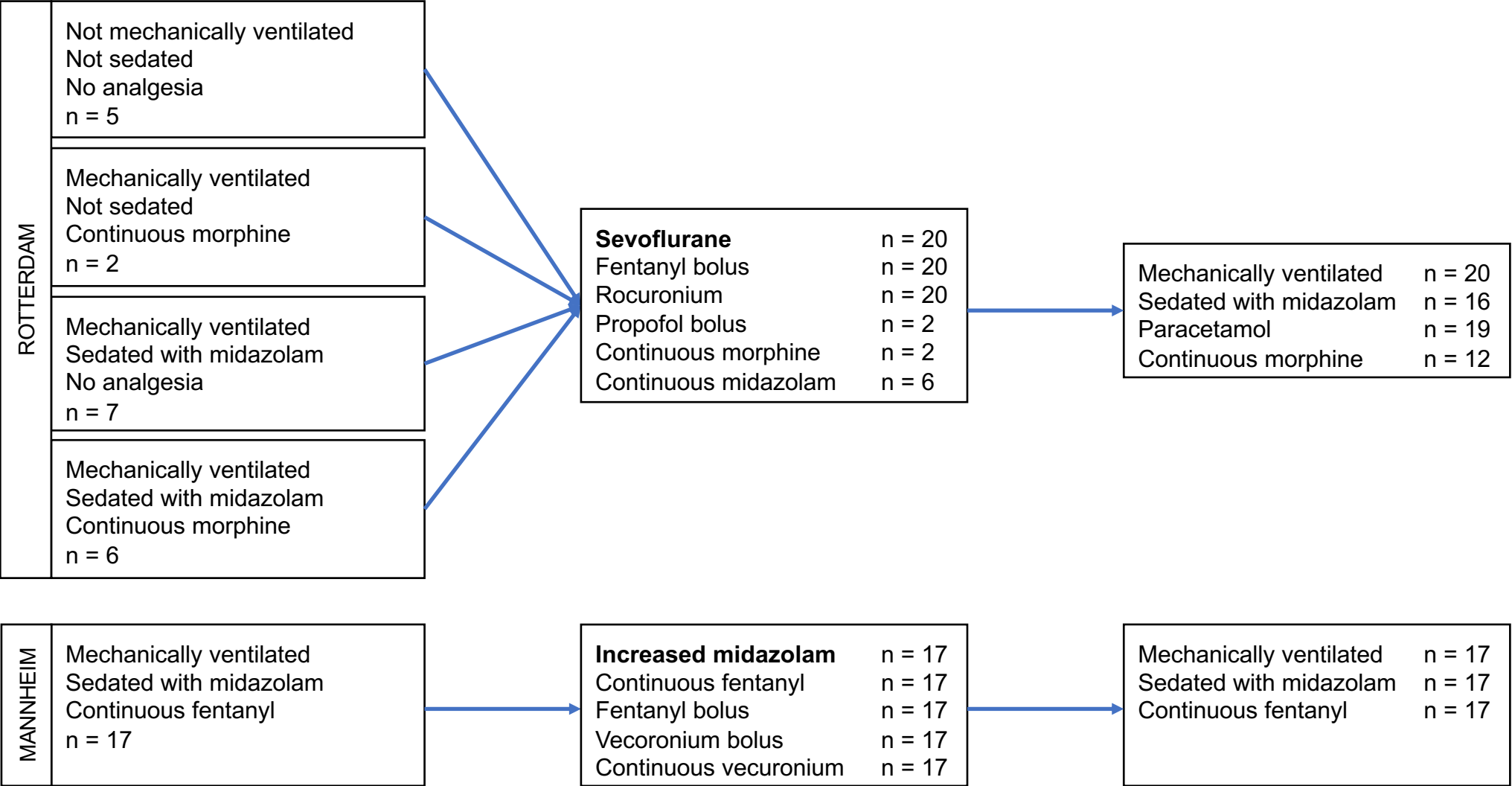

Supplement: Appendix 1 — Flowchart of the included patients. [file Data_Sheet_1.PDF]

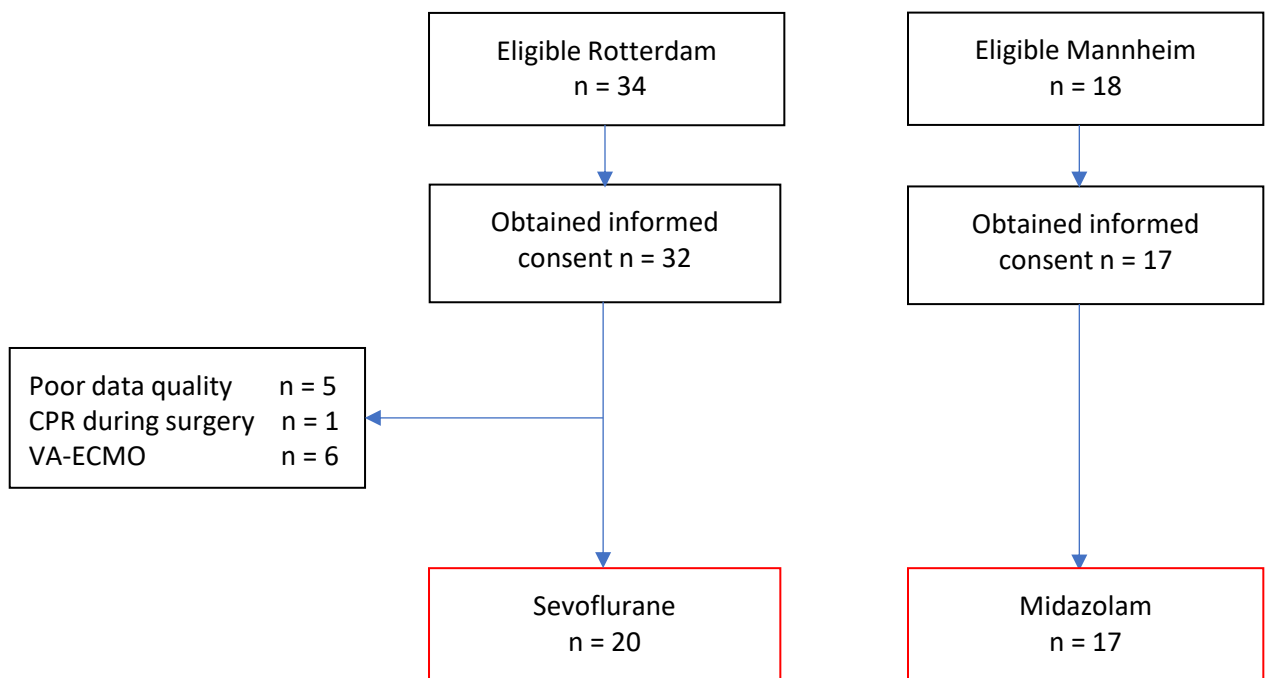

Supplement: Appendix 2 — Overview of administrated medication in the perioperative period. [file Presentation_1.pdf]
