# Supplementary material for: Cerebral Oxygenation and Activity During Surgical Repair of Neonates With Congenital Diaphragmatic Hernia: A Center Comparison Analysis
Source: Front Pediatr. 2021 Dec 17;9:798952. doi: 10.3389/fped.2021.798952 (PMC8718750; doi:10.3389/fped.2021.798952)
Supplement: Appendix 3 — Overview of exact values Pr, In, Po3. [file Table_1.DOCX]

|  | **Sevoflurane group (N = 20)** | | | |  | **Midazolam group (N = 17)** | | | |
| --- | --- | --- | --- | --- | --- | --- | --- | --- | --- |
|  | Pr | In | Po3 | Po15 |  | Pr | In | Po3 | Po15 |
|  |  |  |  |  |  |  |  |  |  |
| HR  (bpm) | 137  126 – 141 | 138  132 – 156 | 127  120 – 135 | 131  127 - 144 |  | 144  138 – 150 | 163  154 – 173 | 141  139 – 149 | 145  138 - 153 |
| MABP  (mmHg) | 48  45 – 51 | 44  42 – 48 | 45  43 – 50 | 47  43 – 52 |  | 50  46 – 53 | 52  49 – 58 | 52  47 – 59 | 52  45 - 53 |
| SpO2  (%) | 98  97 – 99 | 97  96 – 99 | 97  96 – 98 | 98  97 – 99 |  | 98  96 – 99 | 98  97 – 99 | 99  97 – 100 | 99  98 – 100 |
| rScO2  (%) | 81  78 – 89 | 84  77 – 95 | 80  76 – 87 | 79  74 - 85 |  | 76  71 – 86 | 65  61 – 77 | 74  65 – 80 | 73  67 – 78 |
| FTOE  (%) | 17  9 – 20 | 14  5 – 21 | 18  9 – 21 | 19  12 - 25 |  | 0  0 – 12 | 30  2 – 60 | 0  0 – 43 | 0  0 – 44 |
| Time in hypoxia  (%) | 0  0 – 1 | 2  0 – 10 | 0  0 – 2 | 2  0 – 8 |  | 25  11 – 29 | 31  26 – 36 | 22  15 - 32 | 25  16 - 32 |
| EEG $\delta_{1}$ ($\mu V^{2}$) | 7.9  5.5 – 8.6 | 2.2  1.9 – 3.0 | 5.0  4.2 – 6.0 | 6.7  5.6 – 7.6 |  | 4.3  3.5 – 5.7 | 4.3  3.3 – 5.8 | 3.4  2.9 – 5.2 | 3.4  2.6 – 5.9 |
| $\delta_{2}$ | 2.0  1.5 – 2.3 | 0.7  0.6 – 0.9 | 1.4  1.2 – 1.7 | 1.7  1.5 – 2.4 |  | 1.5  1.3 – 2.4 | 1.4  1.0 – 1.7 | 1.3  1 – 1.8 | 1.3  1.0 – 1.8 |
| $\gamma$ | 0.2  0.2 – 0.3 | 0.1  0.1 – 0.1 | 0.1  0.1 – 0.1 | 0.1  0.1 – 0.2 |  | 0.1  0.1 – 0.1 | 0.1  0.1 – 0.1 | 0.1  0.1 – 0.2 | 0.1  0.1 – 0.2 |
|  |  |  |  |  |  |  |  |  |  |
| VIS | 0  0 – 5 | 9  5 – 17 | 2  0 – 11 |  |  | 17  10 – 25 | 17  12 – 35 | 17  10 – 28 |  |
| PaCO2  (kPa) |  | 6.5  5.9 – 7.6 |  |  |  |  | 5.5 4.7 – 6.7 |  |  |
